# Ipecacuanha Instead of Tartar Emetic in Croup

**Published:** 1860-09

**Authors:** J. C. Shapard

**Affiliations:** Flat Creek, Tenn.


### IPECACUANHA INSTEAD OF TARTAR EMETIC IN CROUP.

BY J. C. SHAPARD, M. D., OF FLAT CREEK, TENN.

I am strongly impressed with the opinion that much harm has resulted from the employment of tartar emetic in croup. It is but justice, however, to admit that this opinion is not the result of long experience or extensive observation. And moreover, according to an ancient sage, an opinion is only the half-way house between ignorance and knowledge. I shall content myself with expressing the opinion, without attempting to estimate its value.

I think I have seen fatal prostration result from the use of tartar emetic in infantile cases in my own practice, and in the practice of other physicians; physicians, too, who were regarded as "skillful and scientific," and who deservedly rank high in the profession. That it was prescribed scientifically in the fatal cases alluded to, I have not a doubt. That is, that according to the "teaching" upon the subject it was indicated in those cases. It is the province of our teachers to lead the van of the profession and give the "word of command." They tell us to use tartar emetic in croup; but in justice to them it must also be said they enjoin caution in its administration, and this injunction may not be sufficiently regarded by the subalterns in the professional army.

Among the pathological elements of croup are inflammation, with or without fibrinous effusion, and spasm. Tartar emetic, in sufficient doses will arrest the inflammation, throw off the fibrinous product, and overcome the spasm; but in accomplishing these results a dangerous prostration is much to be feared. And when we remember how many physicians there are who are more inclined to the heroic than the prudent, it becomes a desideratum to find a remedy that will be as efficacious as the tartar emetic, without its dangers. According to M. Petit, ipecacuanha is that remedy.

M. Petit says, in a recent journal, he has been practising medicine in Paris fifty-two years, and that during his long career he has not lost a single case of croup. He treats the disease with emetics—generally ipecacuanha—tartar emetic *never*. He administers the ipecacuanha in syrup, in infusion, or in powder, according to the age or urgency of the case, but always in emetic doses. He prefers the ipecacuanha because he considers it a superior emetic, because it can be administered in large doses with impunity, and for the reason that those who give their attention to the little patient are in no danger, through error or inexperience, of injuring it by heroic practice. He thinks it is the emesis that cures the croup—that the vomitings and efforts to vomit detach and throw off the false membranes—that the general shock, and the perspirations produced by the vomitings, modify the organism in such a manner as to prevent the formation of new false membranes. But, in order to obtain these desirable results, he says it is necessary to insist on the emetic without the fear of fatiguing the little patient, as long as the indications for its use continue, and to return to it after having obtained these results, should the croupal cough and dyspnœa return, without waiting for the respiration to become wheezing. And if the physician wishes to be sure of curing his patient, he should not content himself with prescribing the emetic, but he should administer it himself, or have it administered in his presence; and he should not leave the patient until its respiration is completely relieved, and until it can rest easily in the horizontal position.

M. Petit has met with extraordinary success in the treatment of croup—greater perhaps than most other physicians have obtained from any treatment whatever—even the emetic plan; but his long sustained eminent position is a guarantee of his good faith and scientific attainments, and although others might not be as successful with the ipecacuanha as he has been, yet I think its substitution for tartar emetic would lessen the mortality of croup.—*Nashville Medical Journal.*

